# Modeling Meets Metabolomics—The WormJam Consensus Model as Basis for Metabolic Studies in the Model Organism *Caenorhabditis elegans*

**DOI:** 10.3389/fmolb.2018.00096

**Published:** 2018-11-14

**Authors:** Michael Witting, Janna Hastings, Nicolas Rodriguez, Chintan J. Joshi, Jake P. N. Hattwell, Paul R. Ebert, Michel van Weeghel, Arwen W. Gao, Michael J. O. Wakelam, Riekelt H. Houtkooper, Abraham Mains, Nicolas Le Novère, Sean Sadykoff, Frank Schroeder, Nathan E. Lewis, Horst-Joachim Schirra, Christoph Kaleta, Olivia Casanueva

**Affiliations:** ^1^Research Unit Analytical BioGeoChemistry, Helmholtz Zentrum München, Neuherberg, Germany; ^2^Chair of Analytical Food Chemistry, Technische Universtität München, Freising, Germany; ^3^Epigenetics Department, Babraham Institute, Cambridge, United Kingdom; ^4^Department of Pediatrics, University of California, San Diego, La Jolla, CA, United States; ^5^Centre for Advanced Imaging, The University of Queensland, Brisbane, QLD, Australia; ^6^School of Biological Sciences, The University of Queensland, Brisbane, QLD, Australia; ^7^Laboratory Genetic Metabolic Diseases, Amsterdam UMC, University of Amsterdam, Amsterdam Gastroenterology and Metabolism, Amsterdam Cardiovascular Sciences, Amsterdam, Netherlands; ^8^Signaling Department, Babraham Institute, Cambridge, United Kingdom; ^9^BTI/Cornell University, Ithaca, NY, United States; ^10^Novo Nordisk Foundation Center for Biosustainability at University of California, San Diego, La Jolla, CA, United States; ^11^Research Group Medical Systems Biology, Institute of Experimental Medicine, Christian-Albrechts-University Kiel, Kiel, Germany

**Keywords:** *Caenorhabditis elegans*, metabolic reconstruction, metabolism, metabolomics, metabolic pathways

## Abstract

Metabolism is one of the attributes of life and supplies energy and building blocks to organisms. Therefore, understanding metabolism is crucial for the understanding of complex biological phenomena. Despite having been in the focus of research for centuries, our picture of metabolism is still incomplete. Metabolomics, the systematic analysis of all small molecules in a biological system, aims to close this gap. In order to facilitate such investigations a blueprint of the metabolic network is required. Recently, several metabolic network reconstructions for the model organism *Caenorhabditis elegans* have been published, each having unique features. We have established the WormJam Community to merge and reconcile these (and other unpublished models) into a single consensus metabolic reconstruction. In a series of workshops and annotation seminars this model was refined with manual correction of incorrect assignments, metabolite structure and identifier curation as well as addition of new pathways. The WormJam consensus metabolic reconstruction represents a rich data source not only for *in silico* network-based approaches like flux balance analysis, but also for metabolomics, as it includes a database of metabolites present in *C. elegans*, which can be used for annotation. Here we present the process of model merging, correction and curation and give a detailed overview of the model. In the future it is intended to expand the model toward different tissues and put special emphasizes on lipid metabolism and secondary metabolism including ascaroside metabolism in accordance to their central role in *C. elegans* physiology.

## Introduction

Metabolism is a key mediator of the biological processes underlying living organisms. Metabolic changes are at the frontline of the cellular response to environmental or physiological changes, and altered metabolism is a hallmark and driver of the pathologies accompanying conditions such as aging and cancer (Finkel, 2015). Nevertheless, our understanding of all the complexities of metabolic processes in different conditions remains incomplete. The model organism *Caenorhabditis elegans* is emerging as a key resource for the study of metabolism in multicellular organisms, as while it shares much of its central metabolic pathways with humans, it is easy to culture in laboratory conditions, can be grown in large populations of isogenic individuals in order to study purely environmental differences, and has a short life span enabling rapid longitudinal data acquisition even across multiple generations (Tissenbaum, 2015; Maglioni and Ventura, 2016; Shen et al., 2018).

Metabolomics evaluates the metabolic state of a given sample by measuring the concentrations of a large number of small molecules simultaneously, allowing metabolic differences between different conditions to be evaluated. Advances in metabolomics involve the development of methods and standards to more accurately detect, distinguish and quantify small molecules from a broad range of pathways in increasingly smaller quantities of sample material. In *C. elegans* the currently detectable metabolome encompasses >1,000 distinct metabolites (not counting lipids), but this is continuously evolving, and the estimated size of the full metabolome under ordinary conditions may be even > 10,000 distinct molecules based on recent metabolomics work uncovering new metabolites (Artyukhin et al., 2018).

**Figure 1:**
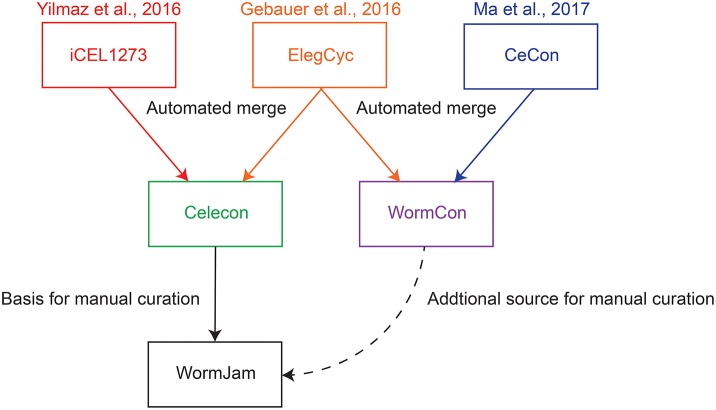
Overview of published *C. elegans* metabolic reconstructions and their relation to consensus models described in this manuscript.

Whole-genome metabolic reconstructions are *in silico* representations of all the metabolic reactions in a given organism as a network of metabolites and the reactions in which they are produced or consumed, with associated genes. They are representations of metabolic knowledge in a given organism abstracted to the level of a single cell. These reconstructions allow sophisticated mathematical analysis techniques to make predictions about the dynamic intracellular fluxes under different conditions. In particular, Flux Balance Analysis (FBA) and its derivatives permit the use of whole-genome reconstructions together with experimental molecular phenotypes and biological objective functions in order to obtain optimal fluxes landscapes at steady-states (O'Brien et al., 2015). One can then observe how these landscapes evolve upon mutations and in different environments, or to predict drug targets and biomarkers. For *C. elegans*, several such metabolic reconstructions exist (Büchel et al., 2013; Gebauer et al., 2016; Yilmaz and Walhout, 2016; Ma et al., 2017) (see Figure 1). We have been working with the whole community to reconcile and develop a single model, representing the best consensus of known metabolism in *C. elegans*. This community effort has been christened “WormJam” (Hastings et al., 2017).

Metabolic reconstructions and metabolomics characterization in different organisms typically proceed independently; the metabolites that are included in a metabolic reconstruction may be different to the metabolites that can confidently be identified in cutting-edge metabolomics investigations in that organism. This is largely due to the different sources of technical complexity and opportunities in the different types of investigation. The gap that ensues between model and metabolomics is one of the specific areas that we are aiming to address with the WormJam effort, which includes participants both from the metabolomics and metabolic modeling communities.

In this paper, we describe the work we have done to develop and extend the consensus model, including merging pre-existing models and curation of novel pathways for *C. elegans*, and the work that is currently ongoing to bridge between the model and the metabolites that can be detected with metabolomics.

## Materials and methods

### Reconciling existing models

#### Merging iCEL1273 and ElegCyc

The merging of these two previously published worm reconstructions ElegCyc and iCEL1273 involved using databases and standards for nomenclature. For genes, we first identified a list of unique genes in each model. ElegCyc (Gebauer et al., 2016) and iCEL1273 (Yilmaz and Walhout, 2016) used different gene identifiers, so a table linking the different gene identifiers for *C. elegans* was obtained (http://geneontology.org/page/download-go-annotations). We chose to work with the WormBase gene identifiers (WBGeneXXXXXXXX) for the sake of their simplicity in parsing and formatting, and accompanying ease of access to the online WormBase database (Lee et al., 2018). If the gene was not found in gene mapping obtained from the GO website, Kyoto Encyclopedia of Genes and Genomes (KEGG) (Kanehisa et al., 2017) was queried to obtain gene information. If duplicate gene entries were found because of different naming conventions used in the model, we updated the gene rules and gene-protein-reaction (GPR) association matrix in the model. A gene-protein-reaction (GPR) matrix is a Boolean matrix identifying genes associated to reaction, a.k.a. gene rules. The gene rules are encoded in a text field which describes two properties about the metabolic reactions: (i) the gene products involved in catalyzing a given reaction, and (ii) the relationship between the gene products involved (isoenzymes—OR, multimers—AND).

For metabolites, we identified a unique list of metabolites using 3 different databases: BiGG (King et al., 2016), KEGG (Kanehisa et al., 2017), and MetaNetX (Moretti et al., 2016). For metabolite identifiers that were in the BiGG database format, we extracted the following information: ID from the Chemical Entities of Biological Interest (ChEBI) (Hastings et al., 2013), KEGG Compound ID (Kanehisa et al., 2017), MetaNetX ID (Moretti et al., 2016), charge, and formula. If a metabolite identifier was in KEGG Compound format, we extracted the following information: name, KEGG compound id/drug id/glycan id, and compound formula. These data were used to map the metabolite to its BiGG identifier and retrieve additional information from BiGG. Information for metabolite identifiers in MetaNetX format were extracted as was done with the BiGG database, and we confirmed the information was consistent by performing a reverse mapping. If neither KEGG nor BiGG information was found, we performed a manual search. Duplicate metabolite entries within the model were fixed by removing one of the instances and resolving in-model meta information and stoichiometry.

**Figure 2:**
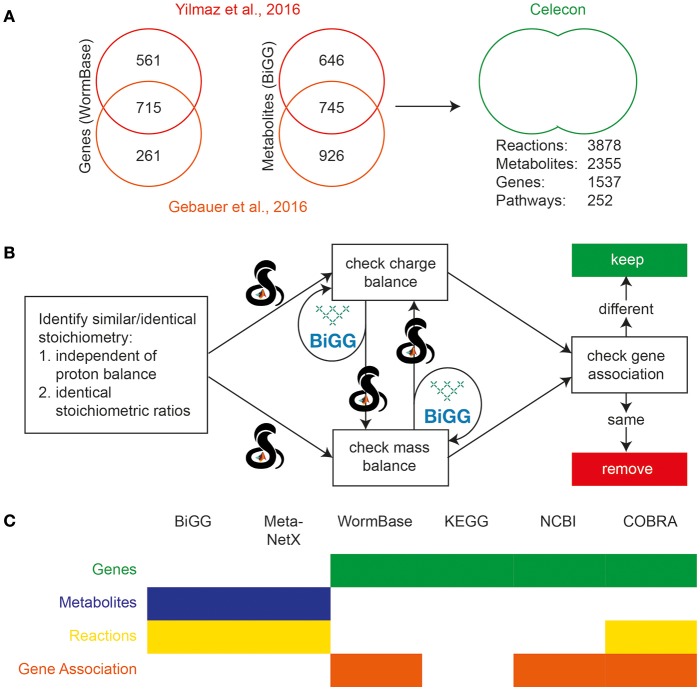
Initial reconciliation and merging of two previously published *C. elegans* reconstructions. **(A)** Genes from the two earlier reconstructions were compared using Wormbase, and metabolites were compared using BiGG. Sizable differences were seen in the scope of the content of the two reconstructions, and so they were curated and merged. **(B)** Duplicate reactions were identified and eliminated using the reaction formulae and information on charge and mass balances using the BiGG and MetaNetX databases. The calculations were performed using the COBRA Toolbox in MATLAB. **(C)** Several databases were used to reconcile various properties of the previous reconstructions. The filled boxes indicate the usage of the database, while color indicates the property reconciled.

For reactions, we employed a script that does the following steps to identify whether the reactions being compared are the same. All used scripts are available from https://github.com/LewisLabUCSD/celegans_reconciliation. We kept the reactions belonging to the first model (R_1_ = 1893) and parsed through the reactions in the second model (r_2_ ∈ [R_1_+1,R_C_] reactions). R_C_ is the total number of reactions including duplicates in both the models. We performed the following steps on each reaction (r_1_ ∈ [1,R_1_]) from the first model:

- Check the *stoichiometry* of ${\text{r}}_{1}^{\text{th}}$ reaction with all reactions (R_1_+1 to R_C_ reactions) (See Figure 2B).
  - If the stoichiometry is the same, check gene-reaction association of the reactions.
    1. If the gene-reaction association is the same, remove the reaction (r_2_) coming from the second model.
    2. If the gene-reaction association is different, append the gene rules in the first model using an OR rule and add the relevant gene associations to the gene-reaction association matrix of the first model, then remove the reaction (r_2_) coming from the second model
  - If the stoichiometry is different, move to r_1_+1^th^ reaction.
- Check the stoichiometry after removing protons to identify if the reactions are the same but have different charge/element balance (See Figure 2B).
  - Perform *elemental balancing* on both reactions to identify the correct version.
  - Repeat steps from (a) again.
  - *Ensure that in-model meta information is consistent*. The in-model reaction meta information is copied from the source model of the reaction; for e.g., if RXNA (has correct balancing) in the second model and is identified as a duplicate of RXNB (has incorrect balancing) in the first model, all in-model meta information will be set to the reaction values from the second model.

#### Merging of CeCon and ElegCyc

To begin merging, ElegCyc was obtained in SBML format from the Supplementary Information of Gebauer et al. (2016), and an SBML format of CeCon (Ma et al., 2017) was exported from Pathway Tools. The two models were then translated into the MetaNetX format (Moretti et al., 2016), and imported into MATLAB for merging. The COMMGEN toolbox (van Heck et al., 2016) contains multiple algorithms for the semi-automated merging of genome scale models, which were applied to the two models. After completely merging the two models into a single COMMGEN entity in MATLAB, the biomass reaction of ElegCyc was set as the objective function for flux balance analysis testing of the model's validity, as CeCon, which was previously used for the mapping of —omic data, does not contain a biomass reaction.

Next, the following steps were performed semi-automatically through COMMGEN functions.

1. Merging of duplicate reactions
2. Merging of reactions with similar species
3. Removal of nested reactions
4. Alteration of invalid transport reactions
5. Removal of invalid external reactions
6. Checking of reactions with the same metabolites, but differing stoichiometry
7. Merging of similar transport reactions
8. Merging of duplicate reactions

In each function, COMMGEN suggested potential merge candidates, and a decision was manually made whether to merge the reactions or keep both based on literature and databases such as KEGG (Kanehisa et al., 2017). After every merge, the model was exported to COBRA Toolbox format, and checked for viability by ensuring flux was able to be carried through the merged model's biomass reaction in a “free-growth” simulation. If the merge was inviable, the change would be reverted. At the end of this process, the resulting model, named WormCon, was exported to SBML format, and further merging was halted in favor of manual curation as part of WormJam.

### Metabolite structure curation

Chemical structures were associated with metabolites included in the consensus reconstruction. For the bulk of the metabolites, structures were found in ChEBI (Hastings et al., 2013). If no structure was available in ChEBI, other databases, e.g., KEGG (Kanehisa et al., 2017), Human Metabolome Database (HMDB) (Wishart et al., 2018), Chemspider (Pence and Williams, 2010), PubChem (Kim et al., 2016), and LipidMaps (Fahy et al., 2009) were queried. Structures not available in any database were drawn in MarvinSketch 18.1.0 (ChemAxon, Budapest, Hungary).

In certain cases, only charged or neutral structures were available in the databases, so the corresponding missing structure was then also drawn in MarvinSketch 18.1.0. All structures missing in ChEBI were submitted via the batch submission tool to assign new ChEBI identifiers (see *Submission of New Structures*).

### Structural similarity calculation

Structural similarity between metabolites was calculated using the Tanimoto similarity of chemical fingerprints as implemented in JChem for Excel 18.5.0196 (ChemAxon, Budapest, Hungary) in Microsoft Excel 2016. To define cut-offs for structural similarity to identify potential reaction pairs, Tanimoto similarities were calculated from known reaction pairs from the WormJam consensus model.

### Secondary identifier, MS/MS, and NMR spectra search

The Chemical Translation Service (http://cts.fiehnlab.ucdavis.edu/) from the Fiehn lab was used to retrieve secondary identifiers from different databases (e.g., KEGG, HMDB, LipidMaps) by the InChIKey if missing. All spectra from MassBank of North America (MoNA, Download 10.June.2018) were downloaded and sorted according to type: LC-MS, GC-MS, CE-MS, *in silico* and others. InChIKey and SPLASH IDs for each spectrum together with the compound name were isolated for comparison against predicted metabolites (Wohlgemuth et al., 2016). NMR spectra were downloaded from HMDB (Download 03.July.2018) and associated with the respective metabolite and its InChIKey.

### Sharing of the reconstruction and manual curation

The first public release of the reconstruction, as described in the current paper can be accessed from BioModels (Chelliah et al., 2015) under the accession MODEL1807230002. To facilitate community curation, the WormJam community uses a shared Google Drive. All past reconstructions and versions of the WormJam reconstruction are stored in standard computer-readable and human-readable formats. To exchange and re-use the models, they are provided in SBML (Hucka et al., 2003), both Level 2 (Hucka et al., 2015) with COBRA-specific notes, and Level 3 (Hucka et al., 2018) with the Flux Balance Constraint package (Olivier Brett and Bergmann Frank, 2018). For manual curation, these models are converted into spreadsheets using a customized SBtab format (Lubitz et al., 2016). Interested persons can join the community by subscribing the WormJam Google group (https://groups.google.com/forum/?hl=en#!forum/wormjam).

### Curation of metabolites from literature

Metabolites detected in different *C. elegans* metabolomics related publications have been manually curated by extracting relevant information from text, tables, figures and Supplementary Information if available. The most unambiguous structure was curated based on details reported in the respective publications. Structural representation in form of SMILES and InChIs were curated together with identifiers from different databases as well as PubMed IDs of the respective publication (Supplementary Information Table 1).

## Results

### Preexisting reconstructions

Several *C. elegans* metabolic reconstructions exist and are used as basis for the WormJam consensus model. The first reconstruction was published in 2013 in the frame of the Path2Models project and was based on automatic reconstruction from KEGG pathways, MetaCyc (Caspi et al., 2016) and a gap-filling step. In 2016, ElegCyc (Gebauer et al., 2016) and iCEL1273 (Yilmaz and Walhout, 2016) were published side-by-side. ElegCyc used PathwayTools (Karp et al., 2016), while iCEL1273 is based on a new workflow developed by the authors. Finally, in 2017 CeCon, also based on PathwayTools, was published by Ma et al. (2017). Table 1 summarizes key metrics of each model.

**Table 1:** Metrics of pre-existing reconstructions.

**Name**	**Compounds**	**Reactions**	**References**	**Notes**
BMID000000141468	3207	2272	Büchel et al., 2013	Automatic reconstruction from KEGG PATHWAYS, MetaCyc and gap-filling
ElegCyc axenic	2357	1893	Gebauer et al., 2016	Bacteria-free growth media
ElegCyc *E. coli*	2357	1921	Gebauer et al., 2016	*E. coli* OP50, *E. coli* biomass composition (Orth et al., 2011) serves as the growth media.
iCEL1273	1718	1985	Yilmaz and Walhout, 2016
CeCon	2166	2085	Ma et al., 2017

To generate a consensus model, different models have been merged together in several stages. The paragraphs below describe the merging process of two reconciliations, which were the basis for the WormJam consensus model (Figure 1).

### Initial automated model merging

#### Merging of iCEL1273 and ElegCyc

The manual curation of the final WormJam model was preceded by finding a consensus and merging two previously published worm models iCEL1273 (Yilmaz and Walhout, 2016) and ElegCyc (Gebauer et al., 2016). The details of these models have been discussed in the previous section. The preliminary merging process was conducted using MATLAB and the COBRA Toolbox. Metabolic models describe a list of reactions which transform chemical compounds (metabolites) using enzymes (gene products). Therefore, we merged the metabolic models by first finding unions of the genes, metabolites, and reactions across all of the models.

##### Genes

We used the list of genes in the GO website (http://geneontology.org/page/download-go-annotations) and KEGG to map common gene names (nomenclature used in the models) to WormBase gene identifiers. We chose to work with the WormBase gene identifiers (WBGeneXXXXXXXX) for the sake of their simplicity in parsing/formatting, and ease of access to the online WormBase database (Lee et al., 2018). For details on reconciliation of genes, please see section Materials and Methods. We were able to map 980 genes in ElegCyc to 974 unique genes and 1292 genes in iCEL1273 to 1276 unique genes.

##### Metabolites

We used four databases: BiGG (King et al., 2016), ChEBI (Hastings et al., 2013), KEGG (Kanehisa et al., 2017), and MetaNetX (Moretti et al., 2016). The metabolite nomenclatures in the models was converted to BiGG as it is the most widely used in the field of metabolic modeling. We used these databases to reconcile various metabolite properties such as charge, formula, and in-model meta information. We were able to map and/or process 1626 metabolites in ElegCyc and 1401 metabolites in WormFlux.

##### Celecon merge (see *Figure 2A*)

The above processing resulted in versions of ElegCyc and iCEL1273 with annotations in the same format including their in-model meta information (i.e., reaction notes, reaction subsystems, reaction compartmentalization, metabolite notes, metabolite charge, metabolite formula, metabolite compartmentalization, database specific identifiers, and/or any other supporting/cross-reference information). Identification of metabolites (M) and genes (G) were followed by performing a merge such that M_C_ = M_1_ + M_2_, G_C_ = G_1_ + G_2_, and R_C_ = R_1_ + R_2_. Here, subscript “1” or “2” represents first or second model, respectively. After identification of duplicate sets of metabolites/genes and combining their respective in-model meta information, we were left with a model with 2355 metabolites (M_C_) and 1537 genes (G_C_) and 3878 reactions (R_C_).

##### Reactions

Following the initial merge, we identified duplicate reactions using steps described in the section Materials and Methods. We used ElegCyc as the first model and iCEL1273 as the second model. The above process was able to remove 611 reactions. The biomass reactions from both the models were kept for evaluation at a later stage. Therefore, the final merged model had 2355 metabolites (M_CycCEL_) and 1537 genes (G_CycCEL_) and 3267 reactions (R'_CycCEL_), with unique lists of gene, metabolites, and reactions with their respective in-model meta information (See Figure 2C).

#### Merging of CeCon and ElegCyc

**Figure 3:**
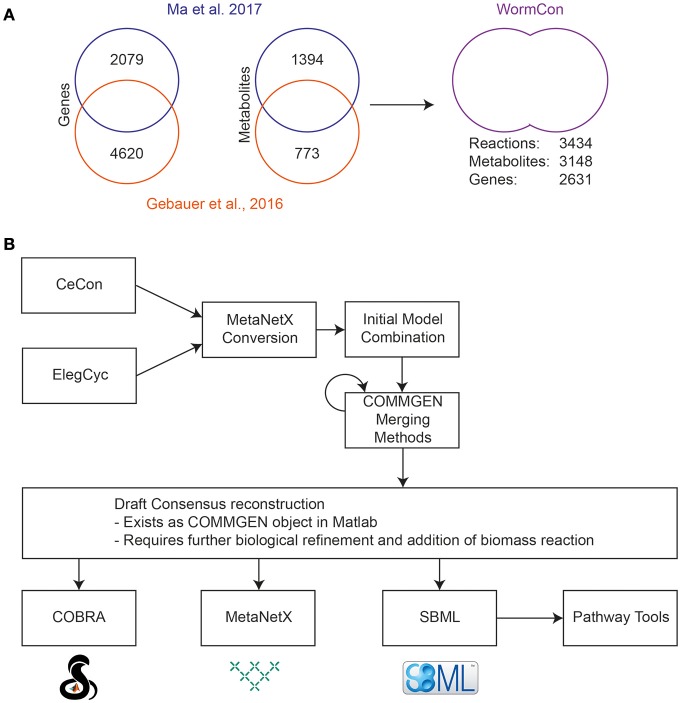
Initial reconciliation and merging of two previously published *C. elegans* reconstructions. **(A)** Metrics obtained from the original Pathway Tools reconstructions of CeCon (Ma et al., 2017) and ElegCyc (Gebauer et al., 2016) and final metrics of the merged model, called WormCon. Data sources for the construction of CeCon and ElegCyc can be found in the original publications. **(B)** The iterative merging process used to generate the draft WormCon consensus model.

WormCon is a consensus model resulting from the merge of CeCon (Ma et al., 2017) and ElegCyc (Gebauer et al., 2016) (Figure 3A). Both models were originally designed in the Pathway Tools software (Karp et al., 2016), and later exported to SBML format. An initial comparison was made between the two models using various top-level metrics including number of reactions and metabolites as a benchmark. These metrics are generated by the Pathway Tools software, and a comparison is shown in Figure 3A. The versions of ElegCyc and CeCon used for merging contained 1193 and 1923 reactions and 773 and 1394 metabolites, respectively. For merging, both models were converted to the MetaNetX namespace, and then combined in MATLAB 2015a using the COMMGEN tool, a package allowing for semiautomatic merging of genome scale models (van Heck et al., 2016). An initial consensus model was automatically generated, then COMMGEN's functions were used to iteratively refine the model as shown in Figure 3B. With the biomass reaction from ElegCyc set as the objective (CeCon does not have a biomass reaction), duplicate reactions were removed from the consensus model. The next two steps were to compare reactions with similar species to determine whether they were the same reaction, just differing by namespace alterations, as well as the removal of nested reactions. Following this, more reactions that could be duplicates were identified. All iterations were performed sequentially, but this resulted in the failing of the model as the merging of reactions vital for the model's function sometimes led to loss of model viability.

To resolve this issue, each stage of merging was performed, then the new version of the model was converted back to SBML, and tested to see if it could produce biomass, and then saved separately if viable. This resulted in a functioning metabolic model, called WormCon can carry flux incorporating reactions from both original models in the process. WormCon contains 2621 genes, 3148 metabolites and 3434 reactions, and has been frozen in its current state for incorporation into the final consensus model.

### Curation of the merged WormJam model

#### Initial manual curation

The starting point for manual curation was the automatically merged Celecon model, with information from WormCon being an additional source for manual curation (Figure 1). Initial manual curation efforts included the further identification and resolution of duplicate reactions that had not been automatically removed (typically because of differences in gene annotation, compartmental localization, or presence/absence of minor cofactors). The charge and ChEBI annotation for metabolites were checked, and duplicate metabolites were also removed (which in some cases led to identification of a set of associated duplicated reactions as well). The metabolic literature was consulted to verify the presence of certain reactions, and in some cases reactions known to be not taking place in *C. elegans* were flagged for removal.

The manual curation effort then further proceeded to enhance and extend the representation of specific aspects of biology not previously included in any of the merged models. This includes the correction of wrong annotations and reactions as well as the addition of new pathways.

#### Correction of pathways

##### Glycogen metabolism

Glycogen metabolism is key to regulate lifespan and healthspan in *C elegans* (Seo et al., 2018). Glycogen is a branched, unbounded, molecule formed by stochastic additions of UDP-glucose molecules forming chains of glucose residues linked by α(1,4) glycosidic bonds from which new chains of glucose residues branch off by α(1,6) glycosidic bonds (Roach et al., 2012). The synthesis is seeded by the protein glycogenin, which also acts as a catalyst for the first glucose extensions. Then glycogen synthase generates the α(1,4) bonds while the glycogen branching enzyme forms α(1,6) bonds. The breakdown of glycogen relies on the split of α(1,4) bonds by glycogen phosphorylase, that produces glucose-1-phosphate and the removal of α(1,6) bonds by the debranching enzyme that produces glucose. Because of the combinatorics born out of the branching and stochastic elongation, it is impossible to model glycogen metabolism accurately and mechanistically. More importantly, because of their complexity and the cycles involved, the metabolism of glycogen in existing reconstructions are often incorrect, consuming, and producing different amounts of glucose residues. Instead, we opted for a phenomenological model that consumes the correct amount of UDP glucose, forms the correct ratio of α(1,4) and α(1,6) bonds, then produces the correct ratio of glucose-1-phosphate and glucose. We also removed the reactions relating to maltose metabolism, as they are likely fossil reactions coming from bacterial metabolism reconstructions. The final pathway is quite simple, with 6 forms of glycogen, and 9 reactions. Each synthesis consumes 12 UDP-glucose, and each breakdown produces 11 glucose-1-phosphate and 1 glucose.

##### Biosynthesis and degradation of fatty acids and BCFA

*C. elegans* is known to produce monomethyl branched chain fatty acids (BCFA) on its own (Perez and Van Gilst, 2008). The different reconstructions only contained lump reactions for this biosynthetic pathway summing up all individual steps into a single reaction. In contrast, the biosynthesis of straight chain fatty acids was represented in much more detail. We have modified the reactions to the same level of detail, which represents always one round of elongation. Likewise, fatty acid oxidation was adapted to the same level of detail.

##### Sphingolipid metabolism

Sphingolipids in *C. elegans* were shown to contain branched chain C17iso sphingoid bases (Chitwood et al., 1995; Zhu et al., 2013; Hannich et al., 2017). However, due to homology searches to human genes, the reconstructions contained C18 sphingolipids produced from condensation of palmitic acid with serine. In contrast to this, *C. elegans* uses C15iso fatty acid which is condensed with serine. We have corrected all sphingolipid metabolites and pathways to contain now the C17iso sphingoid base. Additionally, ceramides in *C. elegans* often contain 2-hydroxy fatty acids. We have added pathways to produce all the 2-hydroxy fatty acids shown to be present in the worm by Chitwood et al., and added ceramide biosynthesis reactions for the normal and 2-hydroxy fatty acids (Chitwood et al., 1995; Hannich et al., 2017).

#### Epigenetics marks

The expression of *C elegans* genome has been shown to be regulated by a range of epigenetics marks, deposited on histones, DNA, or RNA. Some of these marks were present in the starting reconstructions, albeit not always in the right compartment. We fixed histone acetylation, methylation, demethylation, and trimethylation, by adding missing reactions and fixing wrong compartments as well as associated exchange reactions. Similarly, we added a few epigenetic marks recently discovered. They include DNA nuclear N6-deoxyadenine methylation (Greer et al., 2015), modulated by the methylase DAMT-1 and the demethylase NMAD-1, and the ribosomal RNA Cytidine methylation by NSUN5, which has been shown to modulate lifespan (Schosserer et al., 2015). We also added methylation and demethylation of mitochondrial N6-adenine, by *tfbm-1* based on enzyme homology and subcellular localization. These modifications affect different nucleotides and deoxynucleotide residues. We therefore modified the biomass reactions to disentangle the individual bases.

#### New pathways

Different aspects of the worm's metabolism were not covered by previous metabolic models. These pathways and reactions cover *C. elegans* specific metabolic pathways that cannot be inferred from any homology to human genes. We have added two *C. elegans* specific pathways to the model which represent important aspects of the worm's biology.

##### Maradolipids

Maradolipids, chemically 6,6' diacyltrehaloses, have been identified in dauer larvae of *C. elegans* (Penkov et al., 2010). It is suggested that they are required for desiccation tolerance in the dauer stage. Although the exact biosynthetic pathway and genes are not known yet, a potential reaction sequence was added to the reconstruction. Lysomaradolipids, containing only one fatty acid have been described. Therefore, we suggest a stepwise acylation of the two 6 and 6' position of trehalose using fatty acids CoAs as substrates.

##### Ascaroside biosynthesis

Ascarosides are important molecules in the biology of *C. elegans* and serve as messenger molecules in worm to worm communication. For example, different ascarosides are responsible for the entry into the dauer stage upon overcrowding, larval dispersion, and male attraction (Izrayelit et al., 2012; Srinivasan et al., 2012; von Reuss et al., 2012; Panda et al., 2017).

They are produced from long chain (LC) and very long chain (VLC) fatty acids, which are either ω- or ω-1-hydroxylated and attached to ascarylose. In several rounds of peroxisomal β-oxidation, involving the genes *acox-1* (WBGene00008564), *maoc-1* (WBGene00017123), *dhs-28* (WBGene00000991), and *daf-22* (WBGene00013284), these long chain ascarosides are broken down in the form of CoA thioesters to shorter versions (von Reuss et al., 2012).

All metabolic reconstructions developed so far did not contain this biosynthetic pathway, as no corresponding human pathway exists. We have added this pathway by manually curating data from different publications. In a similar fashion, structures of ascaroside-CoA thioesters where drawn and their charged version (−4) was used to construct the peroxisomal β-oxidation pathway. Additionally, hydrolysis reactions to free the individual ascarosides have been added together with export reactions for the extracellular export.

Our current knowledge on the biosynthesis of ascarosides is limited. The involvement of *acox-1, maoc-1, dhs-28*, and *daf-22* in the biosynthesis has been established, which work together for the peroxisomal beta-oxidation of long and very long chain fatty acid versions of ascarosides. Experiments using axenic media have shown that *C. elegans* is still able to produce ascarosides without the presence of bacteria. Therefore, ascarylose biosynthetic genes must be present in *C. elegans*. The exact biosynthetic pathway is not known yet, there are some hints that ascaroside biosynthesis is distinct from the dTDP-rhamnose biosynthesis (Feng et al., 2016). Furthermore, no ascarosyl transferase in *C. elegans* is known. Therefore, a lump reaction generating ascarylose from glucose and adding ω- or ω-1-hydroxyl fatty acids to the sugar moiety was added to the model. Additionally, export reactions from the cytosol to extracellular space were added for the secretion of these molecules. At the current stage only, biosynthetic reactions for basic ascarosides are included. Modified ascarosides have to be added in future versions.

### Current status of the WormJam consensus reconstruction

At present, the WormJam reconstruction encompasses 2833 metabolic compounds distributed across four different compartments—cytosol, mitochondrion, nucleus, and extracellular —, representing 1629 unique chemical species after removing duplicates across compartments. It features 3632 reactions associated with 1524 genes and participated in 70 pathways.

Once the manual curation effort had been completed, the model was rebuilt into computable format from the manually curated spreadsheets in SBtab format for evaluation. Each reaction in the model was tested with respect to its ability to carry flux, and each metabolite whether it could be produced. It is typical for large-scale metabolic models to include some percentage of reactions that cannot carry flux and metabolites that cannot be synthesized. However, it is important that all key pathways are fully functional and biomass can be generated as expected from all cellular precursors. The WormJam model on initial post-curation build contained 1050 reactions that could not carry flux, including the assembly of the primary biomass. However, this was largely due to a few minor errors in key pathways, which were quickly resolved. The current version of the model contains 685 blocked reactions.

### Curation of metabolite structures

#### Representation of metabolite structures in WormJam

The correct representation of metabolite structures is a key issue, since structures represent the most unique identifier for a given molecule. The problem of correct structural representation in metabolic models are two-fold. Firstly, charged molecular structures (and their formulas) representing the major microspecies at a given pH (mostly 7.3 in the cytosol) are required for stoichiometrically correct model representation. This approach is also chemically accurate, since acetate is not acetic acid, they are two different molecules and therefore cannot have the same identifier. However, metabolomics scientists who would like to map their results to metabolic pathways in the model are accustomed to working with neutral formulas and molecules, and directly using charged formulas from the metabolic model to calculate m/z ratios of different adducts leads to incorrect results. In the WormJam consensus model we are using ChEBI as primary source for the structures of charged and uncharged molecules for structural identification. ChEBI represents a rich data source with established chemical ontology relationships to link charged and uncharged variants of different molecules. Therefore, it is essential to also supply the correct neutral form of a molecule that can be used in metabolomics for annotation of signals.

We have added chemical structures encoded as InChIs for each metabolite, where possible. Several metabolites represent generic structures, e.g., “Ceramide” containing an R group for different acyl chain lengths. In such cases we report only the formula containing the R group.

In several cases either the neutral or charged version of the molecules were missing in ChEBI. These have been drawn manually in MarvinSketch 18.1.0 and structures were submitted via the batch submission tool to ChEBI. In total we have submitted >500 new metabolite structures either from the model or the curation of metabolites from literature (see below: *Curation of metabolites from C. elegans metabolomics studies*).

### Metabolites detected in *C. elegans* (metabolomics) studies

#### Curation of metabolites from *C. elegans* metabolomics studies

To get an impression which metabolites have been detected so far in *C. elegans* using different metabolomics techniques and how they map onto the metabolic pathways in the WormJam model, we curated metabolites from over 40 publications. This list is not complete and will be extended in future but represents a starting point to investigate how far our consensus model and metabolomics are diverging.

Metabolites were extracted from the text, figures, and supplementary tables if present (see Supplementary Table 1). The information found, and the level of curation differs in the different articles, ranging from pure names (e.g., leucine) to reporting of metabolite identifiers (e.g., KEGG numbers). To standardize the manual curation of all the publications, we have taken the closest, most probable matching structure from database (mostly from ChEBI). Reporting of ions was normalized to neutral forms, for example acetate was normalized to acetic acid. We used generic structures (e.g., leucine instead of L-leucine) for metabolites where no stereochemistry was reported. In total 1223 unique metabolites were curated for which a structure could be added. Additionally several lipids with generic lipid identifiers such as PC(40:5) were also collected. The total number was 1622 metabolites and lipids detected in 43 publications.

Different analytical techniques (and combinations thereof) were used for analysis of the worm metabolome. Most studies used NMR (14 publications) or NMR in combination with GC- or LC-MS (3 and 6 publications, respectively), followed by LC-MS (9 publications) and GC-MS (5 publications) alone. Furthermore, for lipid analysis, shotgun lipidomics was employed several times. Several metabolites were detected with different techniques, which might increases the confidence of the different identifications. Thirty seven percent of the metabolite structures were detected at least twice, <5% ten times and more. Glutamic acid (CHEBI:18237) and Glycine (CHEBI:15428) were the most frequently detected metabolites, with each being detected 30 times. However, multiple detection and identification have to be handled with great care, since confidence scores or experiments for identification were not always given. It might also happen that wrong annotations spread across several papers once it has been assumed to be positively identified.

#### Comparison of “*in silico*” and measured metabolites

**Figure 4:**
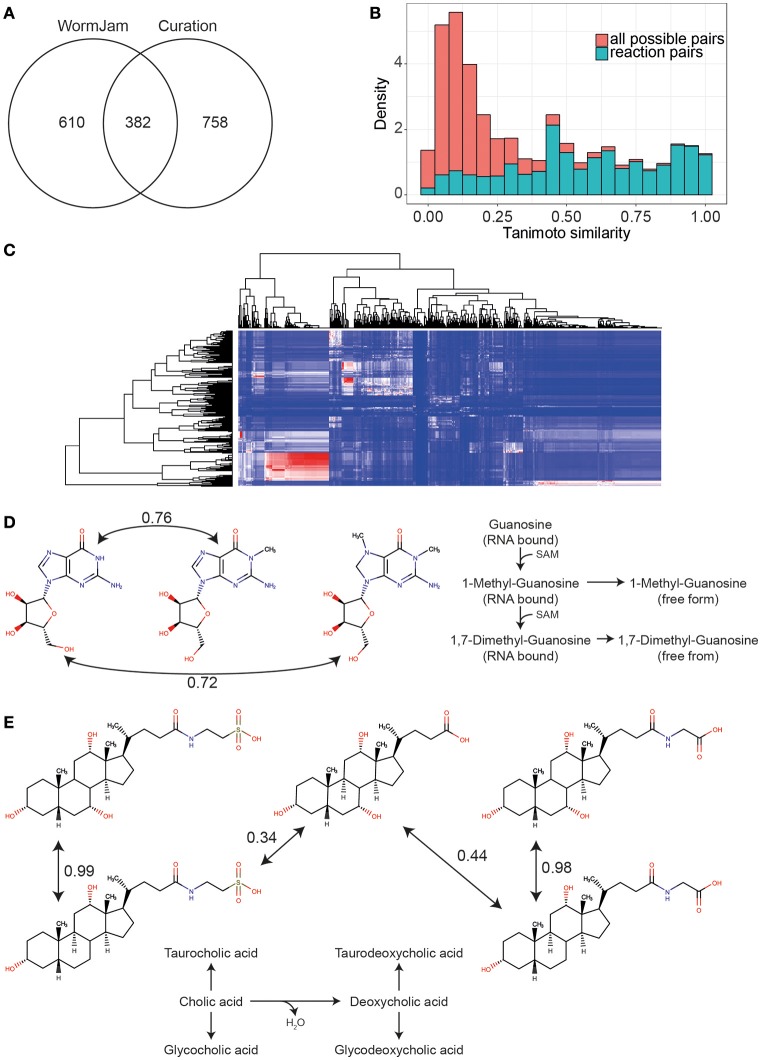
**(A)** Overlap of unique metabolite entities (first part of InChIKey) between the WormJam model and metabolites curated from literature **(B)** Histogram of Tanimoto similarities between all possible metabolite pairs in the WormJam model (red) and metabolites connected by a biochemical reaction (blue) **(C)** Heatmap of Tanimoto similarities between metabolites detected but not in the model (rows) and metabolites from the WormJam model (columns). Color scale is blue for 0 to red for 1. Whereas, 1 refers to a high structural similarity. The large red cluster contains mostly fatty acids and ascarosides. **(D)** Tanimoto similarity between guanosine and 1-Methylguanosine and 1,7-Dimethylguanosine (both detected but not in the model). These metabolites have high structural similarity indicating a potential relation as substrates and products in biochemical reactions. Both the methylated and dimethylated from are derived from RNA bound modified nucleotides, which are released upon degradation. **(E)** Structural similarity between bile acids from the model (cholic acid, glycocholic, and taurocholic acid) and glycodeoxycholic and taurodeoxycholic acid detected in different studies.

To get an impression of the coverage of the *C. elegans* metabolome across all included publications, we compared the percentage of metabolites that have been detected in any publication with the metabolite structures present in the WormJam model. Since the structures reported in the curated publications might not reflect the actual stereochemistry, or the used analysis method cannot correctly determine the stereochemical configuration, we compared the first part of the InChI only, which represents the atom connectivity layer. This yields in total 992 unique entities for the WormJam model and 1140 for the literature curated metabolites. These metabolites were mostly derived from the following metabolite classes: amino acids, fatty acids, central carbon metabolism, lipids, and secondary metabolites like bile acids and ascarosides. A detailed list of curated metabolites can be found in Supplementary Information Table 1. This approach also merges some different structures into a single entity (e.g., β-D-galactose 6-phosphate, NBSCHQHZLSJFNQ-FPRJBGLDSA-N, CHEBI:41076 and β-D-glucose 6-phosphate, NBSCHQHZLSJFNQ-VFUOTHLCSA-N, CHEBI:17719), but allows a fairer comparison of the predicted and detected metabolites by ignoring stereochemistry. Three hundred eighty-two metabolite entities overlapped between the model and the detected metabolites (Figure 4A). Common metabolite entities include organic acids, amino acids, fatty acids, nucleotides, cofactors and several ascarosides.

Interestingly, 758 of the detected entities have no counterpart in the model. We closely examined several of the metabolites. To identify potential connection points to metabolic pathways or metabolites of similar structures in the WormJam model, we used chemical similarity between predicted and detected metabolites not found in the model. Tanimoto similarity was used as measure of chemical similarity (Nobeli et al., 2003). High Tanimoto similarities are found between highly similar chemical structures. Therefore, neighbors in metabolic pathways should show high similarity values. To identify a good cut-off value for the Tanimoto similarity, we first calculated this value for all substrate-product pairs in all reactions of the WormJam model removing hub metabolites like water, ATP, CoA etc. A background dataset with all combinatorial possible metabolite pairs was calculated to determine which values are most informative. Figure 4B shows the histograms for both distributions. Based on this data we decided to use a cut-off >0.4. The pairs contained several generic similarities, e.g., fatty acids are similar to other fatty acids. A large cluster was occupied by fatty acids, sugars and several ascarosides (Figure 4C). Several examples are presented in the following paragraphs related to specific aspects of metabolism or the biology of *C. elegans*. This also includes examples where identifications are in contrast to the current knowledge of *C. elegans* metabolism and more experiments for verification of falsification need to be performed.

One example is the detection of creatine and creatinine. Three of the studies have reported creatine and four creatinine using different analytical methods. No evidence for the use of creatine and creatinine in *C. elegans* exists. The worm uses arginine instead of creatine as phosphagen. *C. elegans* harbors a putative ortholog of the human mitochondrial creatine kinase CKMT2 (W10C8.5, WBGene00021128), however this might also act as arginine kinase. This enzyme was expressed at a higher rate, together with muscle related proteins, in *daf-2* worms (Depuydt et al., 2013), but no ortho- or homologs for the enzymes required for the biosynthesis of creatine have been identified. Creatine and creatinine might have been misidentified.

Several small organic acids have been identified in the exometabolome of *C. elegans*. These acids, including e.g., isovaleric acid or 3-hydroxyisovaleric acid are breakdown products of branched chain amino acids and have been measured in the exometabolome of mitochondrial mutants or anoxic conditions. In the WormJam model, they are only present bound to CoA, despite several carnitine derivatives of these organic acids being detected experimentally. Since these organic acids are produced in the mitochondria they also have to be transported, which is achieved by the carnitine transport system. Small hydroxy organic acids are excreted by long-lived mitochondrial mutants (Butler et al., 2010, 2013).

Interestingly, several fatty acids were not present in the model, plus fatty acid ethanolamides. Both play important roles in the biology of *C. elegans*. Fatty acids represent building blocks of lipids and are also linked to production of secondary messengers and signaling molecules. Related to this class of metabolites, several new ascarosides are contained in the list of metabolites curated from literature. These ascarosides contain very long fatty acid side chains, which are currently not covered in the consensus model. Also, the respective fatty acids (>C26) are not present in the model. Currently, only two publications have detected a C30:0 fatty acid (Gao A. et al., 2017; Gao A. W. et al., 2017). There is growing evidence that the biosynthesis of ascarosides is more complex than previously expected (Zhou et al., 2018). So far, only peroxisomal beta-oxidation of simple ascarosides is covered in the model. Several individual lipid species have been reported.

Several modified nucleotides have been detected in one particular study, including 1-Methylguanosine, 1,7-Dimethylguanosine, 1-Methylinosine, 5-Methylcytidine, and N6-Carbamoyl-L-threonyladenosine. These nucleotides are found in RNA and represent degradation products if found in free form. These modified bases have been measured by LC-MS from RNA samples in *C. elegans* (van Delft et al., 2017). These modifications play important roles in fine tuning RNA function, e.g., tRNA contains several modified nucleotides. Additionally, the production of these modified nucleotides consumes distinct other metabolites, e.g., SAM, and therefore represents an important sink for methyl groups. Therefore, they will need to be added in future versions of the model (Figure 4D).

Two bile acids, taurodeoxycholic, and glycodeoxycholic acid have been detected, but are not included in the WormJam model. The currently best-studied bile acid-like structures are the dafachronic acids (Mahanti et al., 2014; Aguilaniu et al., 2016). However, recent investigations from two WormJam labs have shown that more steroid derived molecules exist in *C. elegans* than previously expected (Frank Schoeder, personal communication, Figure 4E).

Metabolites that are found in the WormJam model but not detected contain mostly CoA derivatives of different molecules or molecules that are not stable enough for detection or only have a short half-life.

#### Public availability of MS/MS and NMR spectra and reference standards

Correct identification of metabolites from non-targeted metabolomics plays an important role for biological interpretation and relies on the availability of reference standards and reference spectral libraries. Therefore, we checked the availability of tandem MS spectra in MassBank of North America (http://mona.fiehnlab.ucdavis.edu/). Spectra were sorted into LC-MS, GC-MS, CE-MS, *in silico* and others (no method available in metadata). Metabolites from the WormJam model were used for the search.

We used the InChIKey as search criteria. First, the complete InChIKey was used to identify perfect matches. Two hundred eighty-eight metabolites matched exactly to LC-MS spectra, while 230 were found to have at least one GC-MS related entry in MoNA. Between the two 199 were overlapping. Metabolites for which reference tandem MS spectra are available are mostly from central metabolic pathways and for which reference materials are readily available.

Likewise, we checked for the public availability of NMR spectra. We downloaded all NMR spectra files from HMDB and isolated the HMDB IDs and added the fitting InChIKeys, which were used as search criteria for the WormJam metabolites. In total 81 metabolites had one or more NMR spectra associated in HMDB.

We were next interested which metabolites are publicly available as reference standards for future metabolomics experiments. We queried the catalog of Sigma-Aldrich/Merck, Cayman Chemicals, and other vendors and checked for availability of substances. Four hundred forty metabolites are available from Sigma-Aldrich/Merck and 131 from additional chemical vendors. For most metabolites for which MS/MS and NMR data is available, a reference standard is also available. Poorly covered areas are different: mostly secondary metabolites, e.g., ascarosides. The Schroeder lab has synthesized a range of reference standards for this substance class, which are also available upon request (Frank Schroeder, personal communication). However, for future metabolite annotation and identification in *C. elegans*, reference spectra will be required in the public domain.

#### Concentrations of metabolites in worms

Although many metabolites have been detected, so far only a few papers report absolute concentrations of known substances. One particular example is presented by Gao A. W. et al. (2017). Results for analysis of fatty acids and amino acids are an exception as they were expressed in nmol/mg of protein. Concentrations from different labs, however might not be directly comparable with each other due to differences in worm cultivation (e.g., different peptone/tryptone). However, a concentration range in which a metabolite might be expected can be derived from this. The curation of this valuable information will become more and more important. Following the example of the human metabolome database, which reports metabolite concentrations in different biofluids for healthy and diseased individuals, concentrations linked with the genotype and phenotype of a worm experiment shall be stored centrally. WormBase represent the ideal candidate for storing this kind of data.

## Outlook

### Overview of the WormJam reconstruction

While the current WormJam reconstruction is a good consensus between existing efforts, and the most accurate *C. elegans* metabolic model to date, the task is by no means completed. The accuracy of the metabolic reconstructions is not uniform across biochemistry. For example, the metabolism of carbohydrates and peptides is well-described, but the coverage of nucleic acids and lipids can be improved. The number of different compartments is limited, and in particular some organelles featuring very specific biochemistry, such as lysosomes and peroxisomes, are not explicitly represented. Moreover, *C. elegans* is a multi-cellular organism interacting with a complex environment, and its cellular metabolism cannot be understood in isolation.

### Integration with diet and omics data

The typical cultivation of *C. elegans* involves maintenance on three possible Escherichia coli strains OP50, HB101, and HT115. Dietary (bacterial/axenic) and nutritional changes have been known to alter health, lifespan (Fontana and Partridge, 2015) and drug efficacy in nematodes. For example, several recent studies in *C. elegans* have shown that nematodes fed with different bacterial strains respond differently to cancer drugs (García-González et al., 2017; Scott et al., 2017). Another recent study showed that serotonin-increased feeding resulted in increased synthesis of aging-related proteins (Gomez-Amaro et al., 2015). In contrast, in its natural environment, *C. elegans* harbors a rich community of bacterial commensals that are distinct from its environmental microbiota indicating a certain level of host selection of bacteria (Dirksen et al., 2016; Schulenburg and Félix, 2017; Zhang et al., 2017). Similar to the microbiota in higher animals, bacteria can have fitness-modulating effects on the nematode including improved growth compared to cultivation on *E. coli* OP50 (Dirksen et al., 2016; Samuel et al., 2016) and a protection against pathogens (Montalvo-Katz et al., 2013). Based on genomic sequences, we have recently started to reconstruct genome-scale metabolic networks of more than hundred bacterial strains found in association with *C. elegans* (Obeng et al., in preparation). Thus, integration of diet is of particular relevance with respect to the utilization of *C. elegans* on *E. coli* OP50 as a model system for microbiome host-interactions in the context of the influence of the microbiome on the efficacy of pharmaceutic therapies (Cabreiro et al., 2013; Scott et al., 2017). We expect that this resource along with the consensus reconstruction of the *C. elegans* metabolism presented in this work and easy experimental amenability will provide a central cornerstone for the establishment of *C. elegans* as a workhorse in the study of host-microbiome interactions.

Biological variability is imminent when an organism is partitioned into different contexts such as environment, mutation, or cell-type/tissue/organ/life stage. Omics data capture these variations in a gamut of biological molecules (Narayan et al., 2016; Cao et al., 2017; Gao A. W. et al., 2017) and thus can be used to generate context-specific models. One of the previous metabolic reconstructions (Gebauer et al., 2016), integrated transcriptomic data to study aging using two previously published algorithms (Shlomi et al., 2008; Zur et al., 2010; Jensen and Papin, 2011). However, there are an array of different algorithms which include but are not limited to mCADRE (Wang et al., 2012), FASTCORE (Vlassis et al., 2014; Pacheco et al., 2016), INIT (Agren et al., 2012), and CORDA (Schultz and Qutub, 2016). The general methodology of these algorithms involves applying constraints on the metabolic network by binarizing the omics data (Richelle et al., 2018). Though there is no clear verdict on which algorithm is most accurate representation of context-specificity (Shlomi et al., 2008; Jensen and Papin, 2011), the need to include a diverse set of omics data to construct context-specific models is apparent. The WormJam model is being curated by taking into account the list of metabolites that have been detected in *C. elegans*, thus facilitating incorporation of context-specific metabolomics data to provide systemic insights.

### Expanding the breadth of understanding of the *C. elegans* metabolome

The refinement of a community network reconstruction of *C. elegans* now provides a context to more comprehensively map out the metabolome. Several further developments will allow us to further grasp this in its full detail. First, the field should strive for better and uniform annotation of metabolites. Efforts such as the WormJam curation will help in this regard, but the actual problem will only be solved once annotation pipelines that are developed in individual institutes will be shared, compared and become publicly available. Second, improving metabolomics techniques for *C. elegans* will improve detection and annotation of metabolites. One example is the annotation of isobaric and isomeric compounds. As for now, these compounds are often difficult to separate using the most widely used metabolomics platforms. Combining conventional mass spectrometry techniques with additional separation techniques such as ion mobility and NMR spectroscopy will allow the identification of such compounds, and aid in improving the annotation. Third, validation: the rapid expansion of the technical arsenal for metabolomics in *C. elegans* calls for caution when it comes to validating methods between labs. Although routine in clinical environments, the same standards should be adopted in a basic research environment. Dense data sets such as those emerging from a metabolomics screen—often involving >1,000 unique metabolites— make it challenging to establish the limit of detection/quantification and assay variability, but this should nevertheless be the ambition to move the field forward. Finally, in addition to the steady state metabolomics protocols it will be important to develop tools to investigate the dynamics of metabolism. Since metabolism is highly dynamic and adaptable, the analyses should reflect this to prevent biased data based on only a single snapshot. Stable isotope tracing allows for such interpretation. Exposing *C. elegans* to for instance ^13^C-labeled metabolic substrates at various time points, in combination with analytical MS, standard NMR; or hyperpolarized NMR spectroscopy (Fan and Lane, 2011; Plainchont et al., 2018) will enable the detection of downstream and intermediate products and reconstruction of the metabolic flux distribution. While this strategy is now widely adopted in mammalian cell-based models, only a few papers describe similar efforts in *C. elegans* (Vergano et al., 2014).

A further important point in the future will be the reporting of confidence in metabolite identification. For our curation we did not include any quality metrics of the metabolite annotation/identification supplied by the authors. However, it will be important to make judgements about the quality of the data. A particular example is found in structures that have not been shown in *C. elegans* before (e.g., creatine and creatinine detected in many different publications). Here identifications might be not correct. Different levels of identification have been proposed by the Metabolomics Standard Initiative (MSI), which should be used to report confidence (Sumner et al., 2007; Haug et al., 2013; Rocca-Serra et al., 2015). Also deposition of data, including identified metabolites and their link to the respective organism, e.g., in Metabolights (Haug et al., 2013), will foster the automatic reconstruction of model organism metabolomes (Salek et al., 2017).

#### Lipids and lipid metabolism

Lipids represent a major bottleneck in metabolic models in general, not only in the *C. elegans* model. Lipids cover a large combinatorial space with different fatty acid combinations possible, which is hard to be represented accurately in a stoichiometric model. In addition, *C. elegans* harbors several peculiarities regarding its lipid metabolism (Witting and Schmitt-Kopplin, 2016). First, it can produce, or acquire from its diet, the full range of saturated, MUFAs, PUFAs as well as odd-branched-chain fatty acids on its own and uses them in all lipid classes. Second, it contains several specific lipid classes like the Maradolipids (Mar) (Penkov et al., 2010), long chain ascarosides and phosphoethanolamine glucosylceramides (PEGCs) (Boland et al., 2017). With the curation of the merged model we have undertaken the first steps toward a better representation. However, major parts are still missing, e.g., better biomass compositions for constraint-based modeling including detailed fatty acid profiles for individual lipid classes. Furthermore, even if detailed biosynthesis pathways for each individual lipid could be integrated into the model, there would still be a huge discrepancy between detailed structure in the model and the annotation and identification capabilities of current lipid analysis methods. With standard lipidomics analysis usually neither the position nor the stereochemistry of double bonds can accurately be identified. Therefore, new ways for modeling, but also for the analysis of lipids need to be investigated to make it possible in the future to incorporate lipidomics data.

## Conclusion

We have shown that there is discrepancy between the model metabolites and metabolites detected by different metabolomics approaches. Specific metabolites from the WormJam model have not been detected so far. Although several are simply not stable enough or have a high turnover rate will be probably never be detected, others might be not accessible with current approaches to low concentrations. On the other side, many detected and reported metabolites could not be found in the model. This is of major interest, since either the identifications are wrong or the model is incomplete. Several examples shown in this paper highlight that we might actually miss several aspects in the metabolism and biology of *C. elegans*. Based on some of our findings the community as whole can now start to explore new metabolic pathways or links to other aspects and their link to metabolism (e.g., epigenetics).

## Author contributions

MWi curated the sphingolipid metabolism, metabolite structures, and metabolites detected in the literature. MWi and FS created an initial draft for the ascaroside biosynthesis pathway. MWa and MvW curated the fatty acid metabolic pathways. AG, MvW, and RH helped with metabolite curation from literature. JPNH created WormCon with the advice of H-JS, PE, and CK, and wrote the relevant manuscript sections on WormCon. H-JS assisted in the development of WormCon, curation of metabolic pathways. PE assisted in the development of WormCon, curation of metabolic pathways and editing the manuscript. CJ, SS, and NL performed the merging of ElegCyc and iCEL1273. JPNH, H-JS, PE, and CK performed the merging of CeCon and ElegCyc. JH, NR, AM, OC, MWi, and NLN performed model validation and have supervised and overseen the manual curation process. All authors wrote and edited the manuscript.

### Conflict of interest statement

The authors declare that the research was conducted in the absence of any commercial or financial relationships that could be construed as a potential conflict of interest.

## Supplementary material

Supplementary Information Table 1 Metabolites curated from literature.
